# The role of d-dimer as first marker of thrombophilia in women affected by sterility: implications in pathophysiology and diagnosis of thrombophilia induced sterility

**DOI:** 10.1186/1479-5876-2-38

**Published:** 2004-11-09

**Authors:** Pierpaolo Di Micco, Maristella D'Uva, Ida Strina, Antonio Mollo, Valeria Amato, Alferio Niglio, Giuseppe De Placido

**Affiliations:** 1 IV Divisione di Medicina Interna e Patologie Epato-Bilio-Metaboliche, Seconda Università di Napoli, Naples, Italy; 2 Dipartimento Universitario di Scienze Ostetriche Ginecologiche e Medicina della Riproduzione, Area Funzionale di Medicina della Riproduzione ed Endoscopia Ginecologica, Università degli Studi di Napoli Federico II, Naples, Italy

**Keywords:** d-dimer, thrombophilia, alteration of haemostasis, sterility, recurrent fetal loss

## Abstract

**Background:**

D-dimer is considered a marker of hypercoagulable state and of endogenous fibrinolysis, so increased d-dimer is detectable in patients affected by thrombosis. Yet, several studies showed that also infertility, in particular secondary infertility due to recurrent fetal losses, has been often related to thrombotic events, in particular in women carrying thrombotic risk factors such as inherited thrombophilia (MTHFR_C677T_, PTHR_A20210G_, Factor V Leiden polimorphisms and/or inhAfter this screening we selected 39erited protein C, protein S, AT III deficiency) or acquired thrombophilia (primary antiphospholipid syndrome, acquired protein C, protein S, AT III deficiency, drugs induced thrombophilia). However, because its high predictive negative value in case of suspected thrombosis, increased d-dimer has been often associated to subclinical thrombophilia. The aim of this study is to investigate the role of d-dimer as first marker of thrombophilia in women affected by unexplained infertility and subsequently to search the cause of increased d-dimer, such as inherited and/or acquired thrombophilia.

**Patients and Methods:**

We selected 79 patients with unexplained primary or secondary infertility. We excluded 40 patients affected by hydrosalpinx, uterine fibroids, uterine malformations, endocrinological and immunological diseases, luteal insufficiency, cytogenetical alterations. All remaining 39 patients were tested for d-dimer and divided in two groups: the patients of group A (25 patients) showed increased plasma d-dimer, in group B were included 14 patients with normal plasma level of d-dimer. After this step all 39 patients were screened for MTHFR_C677T_, PTHR_A20210G_, Factor V Leiden polimorphisms, protein C, protein S, AT III, anticardiolipin IgM and IgG, lupus anticoagulant. In the control group were included 15 age matched women without sterility problems referred to our outpatient's section of vascular medicine for suspected deep venous thrombosis.

Statistical analysis was based on χ^2 ^test, differences were considered to be significant if p < 0.05.

**Results:**

D-dimer was increased in 25/39 and 20/25 showed inherited/acquired thrombophilia while patients with normal d-dimer showed inherited/acquired thrombophilia in 7/14 (p: < 0.05, s).

**Discussion:**

D-dimer is a well known marker of hypercoagulable state, in particular its high predictive negative value in case of suspected thrombosis has been recognised by several reports. Yet, increased d-dimer has been identified also for subclinical thrombophilia besides for vascular thrombosis. Our data, in fact, for the first time suggest an interesting role of d-dimer to identify women affected by unexplained primary or secondary infertility and thrombophilia. So, probably there is a role for d-dimer in these subjects for its predictive positive value. Of course, further data on large based population are needed to confirm our results, because these findings may speed up a diagnostic screening in these patients also for a good cost/effectiveness of this test.

## Introduction

D-dimer is considered a marker of hypercoagulable state besides of endogenous fibrinolysis, so increased d-dimer is detectable in patients affected by arterial and/or venous thrombosis [1]. Yet, several studies showed increased d-dimer also in patients affected by subclinical thrombophilia without ongoing thrombosis [2]. Moreover, also in other clinical conditions, such as chronic inflammation as infectious disease (also as marker of disseminated intravascular coagulation if sepsis is associated) as cancer as necrosis as eldership and pregnancy we may observe an increase of plasma d-dimer [3-8]. So, for this reason d-dimer test is usually used in clinical management for its high predictive negative value in suspected thrombosis, particularly in deep vein thrombosis (DVT) [9-11]. However, several studies showed that frequently women affected by sterility, in particular secondary sterility for recurrent foetal losses, may be affected by an underlying inherited and/or acquired thrombophilia [12-20]. Besides, common thrombotic risk factors which include also a bad lifestyle (e.g. obesity, non regard to Mediterranean diet, sedentary life), a lot of molecular thrombotic risk factors such as inherited or acquired clotting inhibitor deficiency (i.e. protein C, protein S, antithrombin III), inherited thrombophilia (factor V Leiden, prothrombin A20210G mutation), primary or secondary hyperhomcysteinemia, primary or secondary antiphospholipid syndrome and increased plasma factor VIII levels have been identified [21]. Furthermore, these molecular alterations may be also associated in some subjects so inducing gene-gene interactions and/or gene-enviromental interactions [22-24]. So, because the high incidence of clotting abnormalities in these patients, according to the data of Brenner et al. [24,25], we investigated the role of d-dimer as first marker of thrombophilia in women affected by sterility in order to identify causes of increased d-dimer and probably of the induced sterility.

## Patients and Methods

We selected 79 women affected by primary or secondary sterility (due to three or more fetal losses) referred to our sterility center. We excluded 40 patients affected by hydrosalpinx, uterine fibroids, uterine malformations, luteal insufficiency, anovulation, cytogenetical alterations, infectious diseases, endocrinological diseases (ie diabetes, subpituitarism), and by immunological diseases (inherited and/or acquired immunodeficiency, rheumatoid arthritis, systemic lupus erythematosus, systemic sclerosis, vasculitis).

After this screening we selected 39 patients (12 affected by primary sterility and 27 by secondary sterility due to recurrent foetal loss). These 39 patients were tested for d-dimer. D-dimer was measured by several methods [26]; d-dimer were tested randomly in various periods of the ovarian menstrual cycle in 31 patients, in one patient during menstrual bleeding and in seven patients during hormonal therapy in order to obtain controlled ovarian hyperstimulation (COH). Following d-dimer examination patients were divided in two different groups: group A including 25 patients with increased d-dimer levels and group B including 14 patients with normal d-dimer levels. As control group we selected 15 age-matched women, without sterility problem in their anamnesis, referred to our outpatient's section of vascular medicine for suspected deep venous thrombosis.

Subsequently d-dimer evaluation, in order to identify a possible inherited and/or acquired thrombophilia, all patients were screened for methilene-tetra-hydro-folate-reductase C677T gene polimorphism (MTHFR_C677T_), Factor V Leiden gene polimorphism (FVL), prothrombin A20210G gene polimorphism (PTHR_A20210G_), protein S deficiency, protein C deficiency, antithrombin III deficiency (AT III), lupus anticoagulant, IgM and/or IgG anticardiolipin autoantibodies [22,27]. Moreover, all patients showing increased d-dimer were tested also for β-human-corionic-gonadotropin (β-HCG) to exclude early pregnancy, and lower limb ultrasound vascular examination associated to compression ultrasonography (CUS) to exclude a lower limb deep venous thrombosis (DVT); both conditions, in fact, are well known conditions associated to increased d-dimer [4,9-11].

Furthermore, patients with increased d-dimer (group A, 25 patients) and patients with normal d-dimer (group B, 14 patients) were compared also for possible differences in molecular markers of inherited and/or acquired thrombophilia.

Statistical analysis was based on χ^2 ^test, differences were considered to be significant if p < 0.05.

## Results

We found thrombophilia in group A, 80%, and in group B, 50%, so thrombophilia rate in all 39 selected was 65% if we consider together group A (i.e. women affected by sterility and showing increased d-dimer) and group B (i.e. women affected by sterility with normal d-dimer levels) (table 1, 'see additional file 1').

Twenty patients of group A (80%) affected by sterility with increased d-dimer levels, showed inherited and/or acquired thrombophilia [(six MTHFR_C677T _homozygosity, four FVL heterozygosity, five PTHR_A20210G _heterozygosity, three inherited Protein S deficiency, two showing combined defects (one MTHFR_C677T _homozygosity associated to protein S deficiency and one MTHFR_C677T _homozygosity associated to FVL heterozygosity), none protein C deficiency or AT III deficiency, none positive for the presence of lupus anticoagulant, none with increased anticardiolipin autoantibodies IgM and/or increased anticardiolipin autoantibodies IgG)] (table 2, 'see additional file 2'). Remaining five women of the group A did not show molecular thrombophilia, but in their anamnesis we found some possible correlation with an acquired thrombophilia: controlled ovarian hyperstimulation in one patient, monthlies in one patient, early pregnancy in one patient, miscarriage in one patient, none apparent cause in 1 patient; among them two of these five patients were heterozygous for MTHFR_C677T_. Furthermore, two patients of group A carrying inherited thrombophilia for the presence of heterozygous FVL and increased d-dimer revealed previous DVT with following pulmonary embolism in their anamnesis. Data of patients of group A are summarised in table 2 ('see additional file 2').

Seven patients of group B (50%) showed inherited and/or acquired thrombophilia (one MTHFR_C677T _homozygosity, one FVL heterozygosity, five PTHR_A20210G _heterozygosity, none inherited Protein S deficiency, protein C deficiency, AT III deficiency, none presence of lupus anticoagulant, none with increased anticardiolipin autoantibodies IgM and/or IgG), as reported in table 2 ('see additional file 2'). All remaining seven patients of group B showed all heterozygosity for MTHFR_C677T_. Moreover, none patients of group B revealed previous DVT and/or pulmonary embolism.

Five patients of group C (i.e. control group) (33.3%) showed increased d-dimer as molecular markers of ongoing proximal DVT confirmed by ultrasound vascular examination associated to CUS; moreover, all five patients revealed an underlying inherited and/or acquired thrombophilia (three MTHFR_C677T _homozygosity, one protein S deficiency, one combined thrombophilia: FVL heterozygosity associated to protein S deficiency). Data of patients of group C are summarised in table 2 ('see additional file 2').

In all groups positivity for anticardiolipin antibodies or lupus anticoagulant mimicking a primary antiphospholipid syndrome (APS) was not discovered.

So, as showed in table 3 ('see additional file 3'), increased d-dimer is frequently associated with thrombophilia in women affected by sterility, while this association is less present in patients with normal d-dimer, and this difference reaches statistical significance (p < 0.05); furthermore thrombophilia is more frequent in group A than in control group (i.e. group C) and also this difference reaches statistical significance (p < 0.05); finally, thrombophilia in group B is more frequent than in control group (i.e. group C), but this difference does not reach statistical significance (p: 0.08, ns).

## Discussion

In this report for the first time the role of d-dimer was investigated in diagnostic screening of patients affected by sterility and this is a really innovative data available in this clinical setting.

D-dimer is a fibrin degradation product which usually is extensively screened in patients with suspected thrombosis and/or pulmonary embolism [9]. An increased plasma d-dimer might have a predictive positive value for DVT and/or pulmonary embolism, but because increased d-dimer has been observed also in several conditions not associated with ongoing thrombosis (malignancy, chronic inflammation, infections, acute coronary syndromes, necrosis, eldership) [3-9] the really interesting role of d-dimer in this clinical setting is for its high negative predictive value as reported by Bounameaux et al. in a series of patients with suspected pulmonary embolism [9]. However, increased d-dimer has been observed also in subjects affected by thrombophilia (i.e. inherited thrombophilia and/or acquired thrombophilia) showing hypercoagulable state without ongoing thrombosis as reported by Arkel et al. and Humphries et al. [2,23].

So, our data showed that patients of group A, carrying increased d-dimer, has been extensively screened for inherited and/or acquired thrombophilia and 80% of them revealed a well known molecular condition associated to hypercoagulable state which may explain increased d-dimer levels (table 2, 'see additional file 2'). Moreover, this our clinical and laboratory screening reaches statistical significance compared to group B and group C (table 3, 'see additional file 3'). Furthermore, five patients with increased d-dimer did not reveal inherited and/or acquired thrombophilia, but a thorough anamnesis and a clinical evaluation permitted to identify other causes of increased d-dimer in four of these patients: one patient showed early pregnancy (confirmed by β-HCG measurements and following ultrasound scan), a known condition associated to hypercoagulability and increased d-dimer [4,28,29], one patient revealed an early abortion, confirmed by following decrease of β-HCG, and increased dimer levels might be related to uteroplacental thrombosis and/or necrosis [30], one patient was ongoing to controlled ovarian stimulation and this condition may be associated to alteration of haemostasis with a trend toward thrombophilia [31,32] and one patient showed ongoing monthlies, a condition associated to wound healing which involves also clotting factors and might explain increased d-dimer [33]; remaining one patient showed increased d-dimer for unknown causes probably related to not well studied thrombophilia [34] or idiopathic thrombophilia and/or other conditions although we excluded in our selection criteria several other diseases associated to increased d-dimer.

So for the first time we showed an interesting and relevant role of d-dimer in the screening of sterility causes, particular an underlying thrombophilia may be suspected in pathophysiology of sterility if plasma d-dimer is increased. However, also an evaluation of other conditions associated to increased d-dimer (e.g. chronic inflammation, immunopathological diseases, infectious diseases, cancer, necrosis, eldership, pregnancy, controlled ovarian stimulation, monthlies) should be performed in order to avoid a misinterpretation.

Also group B, with normal d-dimer levels, showed an increased rate of thrombophilia (50%, table 1, 'see additional file 1'), so confirming one more time the clear relationship between thrombophilia and sterility, even if these data did not reach statistical significance compared to group C (table 3, 'see additional file 3'). Yet, patients of group B, although affected by thrombophilia and sterility did not show increased d-dimer. This finding might be explained by several causes and a laboratory mistake cannot be excluded; furthermore, these patients of group B may show also transient and/or silent thrombophilia which may trigger a hypercoagulable state if associated to other causes (i.e. acquired conditions associated to thrombophilia) during their natural history and our evaluation of d-dimer might be done during a not-hypercoagulable transient state.

An extensive screening of causes of increased d-dimer in our population was also performed. The association between thrombophilia and sterility due to recurrent foetal loss is well known as reported by several reports [12-20] and also by our data. However, recently an association between primary sterility and thrombophilia has been underlined such as also between thrombophilia and repeated in vitro fertilisation failures [35,36].

A clear relationship between thrombophilia and recurrent foetal loss has been reported for inherited deficiency of clotting inhibitors (i.e. protein C deficiency, protein S deficiency, AT III deficiency) [20,36], but we did not find in our population this strong association (only four cases of protein S deficiency, one of these associated to MTHFR_C677T _homozygosity, and none case of protein C deficiency and/or AT III deficiency). However, this aspect seems to be in agreement with other reports in which other thrombophilic conditions were more frequent than clotting inhibitor deficiencies (e.g. FVL, MTHFR_C677T _homozygosity, antiphospholipid syndrome and so on) [12-20].

FVL gene polymorphism has been frequently found in women affected by recurrent fetal loss, although the frequency of FVL differs in each study [15,24]. These differences could be related, besides to ethnic background, also to different inclusion criteria of investigated patients. However, FVL is associated to sterility also in our study (four cases in group A and one case in group B; table 2, 'see additional file 2').

An increased MTHFR_C677T _homozygosity has been found in our study population (six cases in group A and one case in group B), so confirming a clear role of homocysteine metabolism and of the related hypercoagulable state in sterility pathophysiology [38-40]. Of course, MTHFR gene polymorphism and related homocysteine metabolism may influence sterility also through folic acid and vitamin B12 deficiency due to uncorrected diet and/or lifestyle [41].

We found also an increased frequency of PTHR_A20210G _in women affected by sterility (five cases in group A and five cases in group B), and these data seem to be different from data reported by Pickering et al [42] and Deitcher et al [43] and in agreement with data reported by Brenner et al [24,25]. As we previously underlined, these differences could be related to inclusion criteria established by Investigators of each study and also to an ethnic background; this gene polymorphism, in fact, is more frequent in Southern Europe than in Northern Europe [44,45].

A really interesting data is the absence of APS from our study population and this data differs from data of the Literature. A possible explanation could be offered by different selection criteria: we exclude, in fact, women with immunopathological diseases (e.g. rheumatoid arthritis, systemic lupus erythematosus, systemic sclerosis, vasculitis), so excluding the most common causes of secondary APS and so searching only primary APS that is more rare than primary [46].

In conclusion, in this investigation both groups of women affected by sterility, group A and B, showed increased incidence of thrombophilia compared to control group (group A vs group C, p: < 0.05, s; group B vs group C, p: 0.08, ns; table 3, 'see additional file 3'), so confirming, one more time the relevant role of thrombophilia in pathophysiology of sterility. So, the first relevant data we offer in this study is the role of d-dimer in the screening of sterility causes in order to early suspect an underlying thrombophilia; this screening, as also showed by our data, is in agreement with an elevated frequency of thrombophilia in women affected by sterility (80 % in group A, 50% in group B, 65% if we consider together group A and B). Of course, although several Authors already reported the association between thrombophilia and recurrent foetal loss we may testify that probably the role of thrombophilia is an underestimated problem if we consider all sterility conditions because usually thrombophilia is screened only for repeated foetal loss and not screened in any case of unexplained sterility as in this study.

So, based on our data further studies on large population are needed not only to confirm our results but also to focus a possible different prognosis of these groups, in particular to sterility prognosis.

## Conclusion

Our data demonstrated a clear role of thrombophilia in patients affected by sterility, but suggesting a clear diagnostic role of increased d-dimer in a lot of these patients. This diagnostic screening of thrombophilia in women affected by sterility, based on the d-dimer levels, may also represent a really speed method to suspect thrombophilia in these subject and has also a good cost/benefit ratio, although other causes of increased d-dimer should be always considered. In a second step, if increased d-dimer levels are present causes of hypercoagulable state may be investigated (i.e. inherited thrombophilia and/or acquired thrombophilia). This approach may play a role not only in differential diagnosis of sterility but also in the early diagnosis of sterility due to thrombophilia. After the first step in which d-dimer may be evaluated, causes of increased d-dimer should be subsequently identified in order to start a possible antithrombotic treatment soon.

Nevertheless thrombophilia may be present in few cases also in subjects with normal d-dimer, it should be investigated always if other causes of sterility are not present.

So, we strongly suggest to test d-dimer in patients affected by sterility as first step of a possible underlying thrombophilia in order to early identify the cause of thrombophilia and its prompt treatment but other data should be confirmed by further investigations on large based population.

## Supplementary Material

Additional File 1 Thrombophilia frequency in studied groups Click here for file

Additional File 2 Thrombophilia in studied groups Click here for file

Additional File 3 statistical analysis according with χ^2 ^method Click here for file
