# Supplementary material for: The role of d-dimer as first marker of thrombophilia in women affected by sterility: implications in pathophysiology and diagnosis of thrombophilia induced sterility
Source: J Transl Med. 2004 Nov 9;2:38. doi: 10.1186/1479-5876-2-38 (PMC535536; doi:10.1186/1479-5876-2-38)
Supplement: Additional File 1 — Thrombophilia frequency in studied groups [file 1479-5876-2-38-S1.doc]

***Table 1. Thrombophilia frequency in studied groups.***

| Group | % |
| --- | --- |
| Group A (20/25) | 80 |
| Group B (7/14) | 50 |
| Group C (5/15) | 33.3 |
| Group A plus group B (20/25 + 7/14) | 65 |
