# Supplementary material for: The role of d-dimer as first marker of thrombophilia in women affected by sterility: implications in pathophysiology and diagnosis of thrombophilia induced sterility
Source: J Transl Med. 2004 Nov 9;2:38. doi: 10.1186/1479-5876-2-38 (PMC535536; doi:10.1186/1479-5876-2-38)
Supplement: Additional File 2 — Thrombophilia in studied groups [file 1479-5876-2-38-S2.doc]

**Table 2. Thrombophilia in studied groups.**

| Patients | Group A (25) | Group B (14) | Group C (15) |
| --- | --- | --- | --- |
| Factor V Leiden heterozygosity | 4 | 1 | 0 |
| PTHR_A20210G_ heterozygosity | 5 | 5 | 0 |
| MTHFR_C677T_ homozygosity | 6 | 1 | 3 |
| Protein S deficiency | 3 | 0 | 1 |
| MTHFR_C677T_ homozygosity and Factor V Leiden heterozygosity | 1 | 0 | 0 |
| MTHFR_C677T_ homozygosity and protein S deficiency | 1 | 0 | 0 |
| Factor V Leiden heterozygosity and Protein S deficiency | 0 | 0 | 1 |
| Anticardiolipin IgM and/or IgG | 0 | 0 | 0 |
| Lupus anticoagulant | 0 | 0 | 0 |
| Protein C deficiency | 0 | 0 | 0 |
| AT III deficiency | 0 | 0 | 0 |

Figure legend:

MTHFR_C677T_: methylene-tetra-hydro-folate-reductase C677T gene polimorphism

PTHR_A20210G_: prothrombin A20210G gene polimorphism
