# Supplementary material for: The role of d-dimer as first marker of thrombophilia in women affected by sterility: implications in pathophysiology and diagnosis of thrombophilia induced sterility
Source: J Transl Med. 2004 Nov 9;2:38. doi: 10.1186/1479-5876-2-38 (PMC535536; doi:10.1186/1479-5876-2-38)
Supplement: Additional File 3 — statistical analysis according with χ2 method [file 1479-5876-2-38-S3.doc]

**Table 3. Increased d-dimer and thrombophilia, statistical analysis according with χ^2^ method.**

|  | p |
| --- | --- |
| Group A versus group B | <0.05, s |
| Group A versus group C | <0.05, s |
| Group B versus group C | 0.08, ns |
